# Outcomes and prognostic factors of patients treated for in-stent restenosis: a retrospective single-center experience

**DOI:** 10.1186/s43044-022-00281-x

**Published:** 2022-05-21

**Authors:** Anis Ghariani, Mohamed Aymen Ben Abdessalem, Khalil Cheikh Sideya, Ahmed Fekih Romdhane, Zied Ben Ameur, Hamza Mosrati, Hatem Bouraoui, Abdallah Mahdhaoui, Gouider Jeridi

**Affiliations:** 1 Department of Cardiology, Farhat Hached University Hospital Center, Sousse, Tunisia; 2 Research Laboratory LR14ES05 of Cardio-Pulmonary System Interactions, Ibn El Jazzar Medical Faculty of Sousse, Sousse, Tunisia

**Keywords:** Coronary restenosis, Prognosis, Percutaneous coronary intervention, Stents

## Abstract

**Background:**

The incidence of in-stent restenosis (ISR) remains relatively common despite the use of drug-eluting stents. Outcomes and prognostic factors following ISR revascularization are still being investigated. We aimed to describe the outcomes following different ISR treatment strategies in order to identify prognostic factors associated with worse outcomes.

**Results:**

In a retrospective cohort study, we included patients who were admitted to our department and treated for ISR, from January 2017 to December 2018. All patients were followed up for a median period of 24 months. Major cardiac adverse event (MACE) was a composite outcome of the following events: myocardial infarction, target vessel revascularization, target lesion revascularization or cardiovascular death. MACEs were collected during follow-up. Our population consisted of 116 patients. Mean age was 60 years old with a sex ratio of 2.8. During follow-up, 44 patients (37.9%) had at least one MACE. Independent factors identified by multivariate logistic regression were ISR of the proximal left anterior descending artery [Odds ratio (OR) = 1.29; 95% confidence interval (95% CI) 1.16–1.81; *p* = 0.05], diffuse ISR [OR = 2.16; 95% CI 1.1–3.47; *p* = 0.022], double or triple vessel disease [OR = 2.97; 95% CI 1.2–6.8; *p* = 0.008], two or more stents per lesion [OR = 1.82; 95% CI 1.14–2.21, *p* = 0.031] and absence of post-dilatation in the initial angioplasty [OR = 1.32; 95% CI 1–1.35; *p* = 0.04].

**Conclusions:**

Our study suggested that ISR is related to poor outcomes. Identifying prognostic factors would play a key role in the refinement of interventional techniques.

## Background

In-stent restenosis (ISR) is “the gradual renarrowing of a stented coronary artery lesion from arterial damage with subsequent neointimal tissue proliferation” [1]. The advent of coronary drug-eluting stents (DES) has decreased the incidence of coronary artery ISR [2]. However, it is still encountered in 5–15% of percutaneous coronary interventions (PCI) [3]. Actually, as technologies evolve, more patients with complex lesion characteristics are treated with PCI. Moreover, in our context, bare-metal stents (BMS) are still widely used. Our public health insurance system does not reimburse DES for every PCI. As a result, ISR incidence remains considerable.

National registries of ISR are lacking. Furthermore, few studies have been conducted in our population; especially after the treatment of ISR. Identifying prognostic factors following ISR treatment would play an important role in the improvement of interventional techniques and the choice of revascularization strategies.

Under these circumstances, we sought to investigate outcomes following different ISR treatment strategies in order to identify prognostic factors associated with worse outcome.

## Methods

It was a retrospective cohort study conducted from January 2017 to December 2018 in the cardiology department of Farhat Hached university hospital center. Patients with a history of PCI admitted for recurrent chest angina were eligible for this study. We included patients with a confirmed angiographic diagnosis of ISR currently defined at > 50% stenosis of a previous stented segment or up to 5 mm from the stent edges [4]. Patients with stent thrombosis or already treated for ISR were not included. Patients lost to 1-year follow-up were excluded. Ethical approval was obtained from Ibn El Jazzar Medical Faculty of Sousse ethic committee. An informed and written consent was obtained from each patient. Baseline characteristics and initial procedural aspects were collected from medical files. Baseline characteristics included: age, sex, cardiovascular risk factors (diabetes mellitus, hypertension, active smoking or stopped for less than 3 years, low-density lipoprotein (LDL) cholesterol level higher than 1.4 mmol/l and family history of coronary artery disease), comorbidities (chronic kidney disease with a creatinine clearance less than 60 ml/min and heart failure) and statins therapy. Atorvastatin at 80 mg once daily or Rosuvastatin at 20 mg once daily were considered as high-dose statins therapy.

About initial procedural aspects, diameter, number and type of stents (DES or BMS) were collected. Post-dilatation and procedural complications (iatrogenic coronary artery dissection and significant residual stenosis) were recorded. Angiographic lesions were analyzed by two-dimensional quantitative coronary angiography (2D-QCA) and reassessed by the interventional cardiologist. For ISR clinical and angiographic characteristics, delay for the diagnosis of ISR, coronary status, site and Mehran classification of ISR [5] (I: focal < 10 mm, II: diffuse, III: proliferative or IV: total occlusion) were collected. The choice of ISR revascularization strategy (PCI, coronary artery bypass graft surgery (CABG) or medical treatment only) was left to the interventional cardiologist. If a CABG seemed to be suitable, the decision would be made after the consultation of the heart team.

Patients were followed up for at least 12 months. Major adverse cardiac events (MACE), defined as the occurrence of myocardial infarction, target vessel revascularization, target lesion revascularization or cardiovascular death, were collected during follow-up. For statistical analysis, categorical data were presented as counts and proportions (%). Continuous data were presented as median or as mean ± standard deviation, as appropriate. Differences between groups were evaluated using the Student t tests for continuous data. Chi-squared or Fisher exact tests (if the expected cell value was under 5) were used for categorical variables. A log-rank type test (Mantel-Cox) to compare survival distribution according to each ISR treatment strategy was used. Clinical, angiographic and procedural factors associated with the occurrence of MACEs during follow-up were identified by univariate analysis. Independent factors were then identified by multivariate logistic regression with a stepwise approach. Odds ratio (OR) and confidence intervals at 95% (95% CI) were calculated. All probability values were two sided and considered statistically significant if *p* < 0.05.

## Results

### Patients baseline characteristics

From January 2017 to December 2018, 542 patients with a history of PCI were admitted for recurrent chest angina. Among them, 357 (65.9%) were previously treated with DES and 185 (34.1%) were treated with BMS. Confirmed ISR was noted among 116 patients (21.4%): 64 patients (17.1%) previously treated with DES and 52 patients (28.1%) previously treated with BMS. Mean age was 60 ± 8.5 years old. Male patients represented 74% of the study population with a sex ratio of 2.8. Table 1 illustrates baseline characteristics.

**Table 1:** Patients baseline characteristics

Characteristics	Total *n* = 116 (%)
Sex, male	86 (74%)
Cardiovascular risk factors	
Diabetes mellitus	64 (55.2%)
Hypertension	72 (62.1%)
Smoking (or stopped for less than 3 years)	70 (60.3%)
LDL-cholesterol > 1.41 mmol/l	50 (43.9%)
Family history of coronary artery disease	13 (11.2%)
Comorbidities	
Chronic kidney disease (creatinine clearance < 60 ml/min)	47 (40.5%)
Heart failure	9 (7.7%)
Statins therapy	
High-dose statins therapy*	32 (27.6%)

*LDL* low-density lipoprotein, *LVEF* left ventricular ejection fraction

*Atorvastatin 80 mg once daily or Rosuvastatin 20 mg once daily

### Technical aspects of the initial angioplasty

Nineteen patients (16.4%) had more than two stents in the site of the ISR. The diameters of stents varied from 2.25 to 4 mm. The median diameter was 3 mm. All of DES were second generation and third generation. Everolimus-eluting stents were used for 31 patients (26.7%) of the cases, followed by Sirolimus-eluting stents: 19 patients (16.3%) and Zotarolimus-eluting stents: 4 patients (3.4%). Third generation bioabsorbable polymer stents were used for 17 patients (14.6%). Post-dilatation in the initial angioplasty was performed with 29 patients (25%). We reported 3 cases (2.6%) of iatrogenic coronary artery dissection, 5 cases (4.3%) of no-reflow and 6 cases (5.2%) of significant residual stenosis.

### Clinical and angiographic characteristics of ISR

Mean delay between ISR diagnosis and initial angioplasty was 10.8 months with a median of 7 months. Early ISR, occurring in less than 6 months, was noted among 30 patients (25.9%). We noticed that 53 ISR (45.7%) occurred in the left anterior descending artery (LAD), mainly in the proximal LAD: 25 cases (21.6%). The right coronary artery was the site of 40 ISR (34.5%) while the circumflex artery and the left main were concerned in 18 (15.5%) and 5 (4.3%) cases, respectively. Diffuse ISR was the most frequent presentation: 52 patients (44.8%) while focal ISR concerned 40 patients (34.5%). Proliferative ISR occurred among 19 patients (16.4%) and total occlusive ISR were recorded in 5 patients (4.3%). Forty-six patients (39.7%) had a single vessel disease, 40 patients (34.9%) had a double vessel disease and 30 patients (25%) had a triple vessel disease.

### ISR treatment strategy

Trans-radial access route was used among 72 patients (62.1%). ISR adopted treatment modalities were as follows: 85 patients (73.3%) were treated with PCI; DES was used with 48 patients (41.4%) and drug-eluting balloon was used with 37 patients (31.9%); fifteen patients (13.8%) had undergone CABG and sixteen patients (12.7%) had received medical treatment only.

### Outcomes of treated ISR

**Fig. 1:**
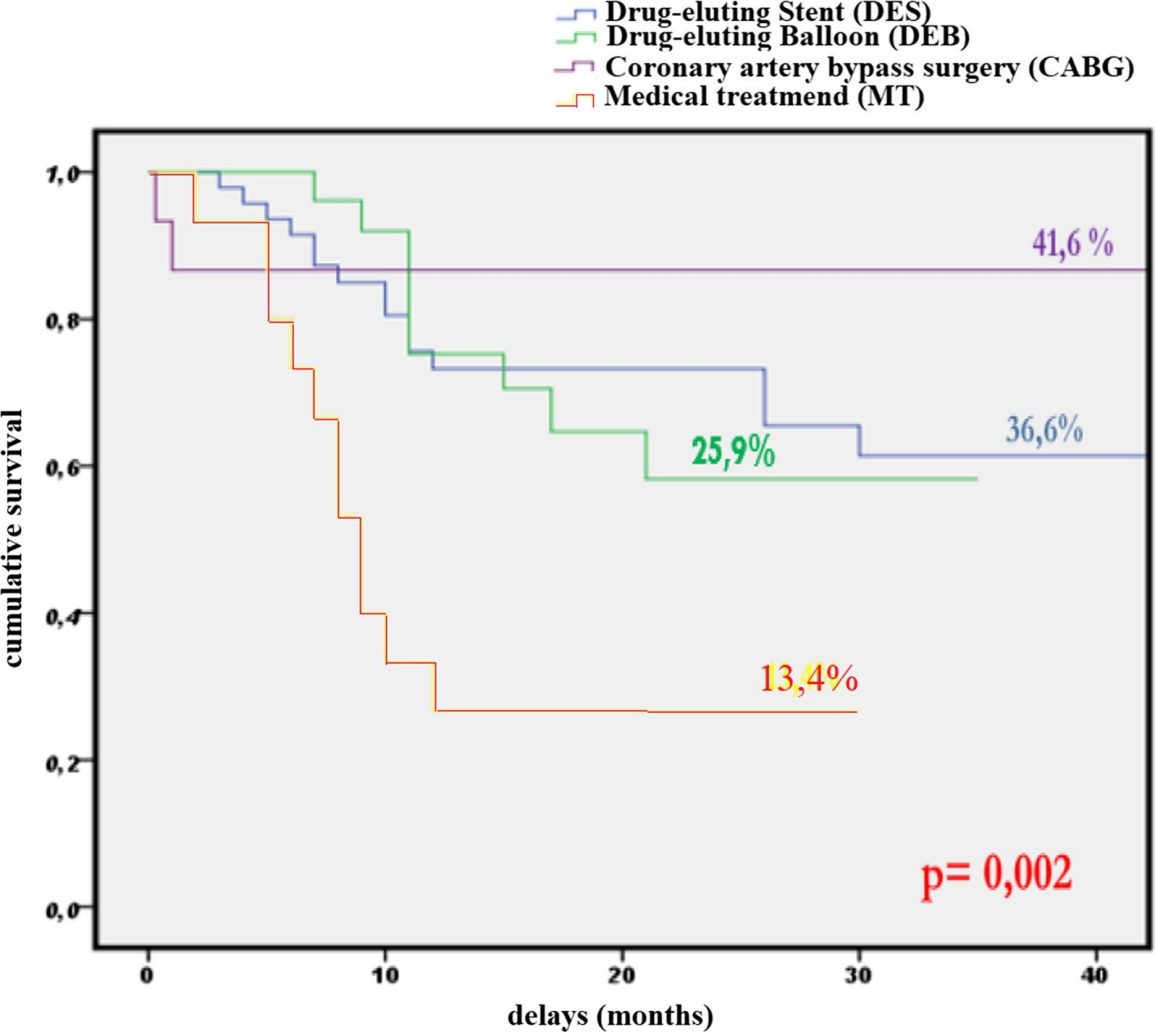
Major adverse cardiac events free survival according to in-stent restenosis treatment strategy. Kaplan–Meier curves represent major adverse cardiac events (MACE)-free survival according to in-stent restenosis treatment strategy. Patients, who received medical treatment only, had the lowest MACE-free survival rate (13.4%). On the contrary, patients, who underwent coronary artery bypass graft surgery (CABG), had the best MACE-free survival rate (41.6%). They were followed by patients treated with percutaneous coronary intervention (Drug-eluting stents: 36.6% and drug-eluting balloon: 25.9%)

All of our patients were followed up for a median period of 24 months [1–51 months]. MACEs were reported among 44 patients (37.9%) as resumed in Table 2. We reported similar MACEs rates following the use of DES and DEB: 33.3% and 40.5%, respectively, *p* = 0.468. We reported MACEs in two patients only among 16 who had undergone CABG, which is significantly less compared with patients treated with PCI (DES or DEB) (12.5% vs. 36.5%, *p* = 0.044). MACEs rates were higher in the group of patients who received medical treatment alone compared with patients treated with PCI or CABG (73.3% vs. 29.7%, *p* < 0.001). Figure 1 represents MACE-free survival rates according to ISR treatment strategy. A log-rank type test (Mantel-Cox) showed a significant difference of survival following different ISR treatment strategy with *p* = 0.002 (Table 3).

**Table 2:** Major adverse cardiac events during follow-up

Event	*N* (%)	Occurrence delay (months) mean/median ± SD [min–max]
Total MACEs	44 (37.9%)	15/17 ± 11.9 [1–51]
Myocardial infarction (MI)	30 (25.9%)	20.5/18 ± 11.9 [2–51]
Target vessel revascularization (TVR)	5 (4.3%)	22.5/24 ± 10.9 [1–51]
Target lesion revascularization (TLR)	24 (20.9%)	20.4/24 ± 11.9 [1–51]
Deaths	11 (9.5%)	22.9/24 ± 10.9 [1–51]

*MACEs* major adverse cardiac events, *max* maximum months, *min* minimum months, *SD* standard deviation

**Table 3:** Log-rank type test to compare survival according to each ISR restenosis treatment modality

	Khi-deux	ddl	*p* value
Log-rank (Mantel–Cox)	16,844	4	0.002

### Prognostic factors associated with worse outcome

Factors associated with higher rates of MACEs during follow-up are resumed in Table 4. They were divided into four groups: clinical characteristics, angiographic ISR characteristics, technical aspects of the initial angioplasty and ISR treatment strategy. Independent factors identified by multivariate logistic regression were ISR of the proximal LAD [OR = 1.29; 95% CI 1.16–1.81; *p* = 0.05], diffuse ISR [OR = 2.16; 95% CI 1.1–3.47; *p* = 0.022], triple or double vessel disease [OR = 2.97; 95% CI 1.2–6.8; *p* = 0.008], two or more stents per lesion [OR = 1.82; 95% CI 1.14–2.21; *p* = 0.031] and absence of post-dilatation in the initial angioplasty [OR = 1.32; 95% CI 1–1.35; *p* = 0.04].

**Table 4:** main predictors of major adverse cardiac events during follow-up identified with univariate analysis

	% of MACEs	*p* value
Clinical characteristics		
Hypertension	58% versus 23.2%	0.049
LDL-cholesterol > 1.4 mmol/l	38.7 versus 18.9%	0.02
Absence of high-dose statins therapy*	17.9% versus 5.48%	0.03
Angiographic ISR characteristics		
Early ISR (< 6 months)	18.1% versus 6.4%	0.042
ISR of the proximal LAD	14.6% versus 8.9%	0.032
Diffuse ISR	31% versus 10.3	0.05
ISR occurring on a BMS	32.7% versus 20.6%	0.03
Coronary status: triple or double vessel disease	17.8% versus 4.8%	0.016
Technical aspects of the initial angioplasty		
Two or more stents per lesion	41.5% versus 19.1%	0.045
Absence of post-dilatation	25.8% versus 3.4%	0.037
ISR treatment strategy		
Medical treatment only	18.8% versus 8.1%	0.046

*BMS* bare-metal stent, *CABG* coronary artery bypass graft surgery, *DEB* drug-eluting balloon, *DES* drug-eluting stent, *ISR* in-stent restenosis, *LAD* left anterior descending artery, *MACE* major adverse cardiac events, *vs* versus

*Atorvastatin less than 80 mg once daily or Rosuvastatin less than 20 mg once daily

## Discussion

### Frequency of ISR

In our study, the frequency of ISR with BMS was 28.1% versus 17.1% with DES. In the European BENESTENT study [6] and the North American STRESS study [7], rates of angiographic ISR at 6 months were 22% and 31.6%, respectively. In the EPISTENT study [8] using more recent stenting techniques, the 6-month angiographic restenosis rate was 8.7%. Morice et al. [9], Windecker et al. [10], and Stone et al. [11] found an ISR rate of 10%, after using DES. In a recently published study, Moussa et al. [12] explored trends and outcomes of ISR and found incidences of ISR at 5% for DES and 16% for BMS. Our results showed slightly higher rates of ISR with both DES and BMS compared to recent studies. Actually, patients treated with DES in our department have higher cardiovascular risk factors especially diabetes mellitus. Compared with other studies, we found that our population is younger, but with a higher frequency of cardiovascular risk factors, mainly diabetes mellitus. Besides, our population has a higher number of chronic kidney disease and heart failure. This can, in part, explain the higher frequency of ISR in our population.

### Mid-term outcomes of treated ISR

In our study, MACEs were reported among 44 patients (37.9%). MACEs rates during follow-up were similar to DES and DEB (33.3% and 40.5%, respectively, *p* = 0.468). Kawamoto et al. [13] also found comparable rates of MACEs following the treatment of ISR with DES and DCB. One-year and two-year MACEs rates were, respectively: DES: 14.0% versus DCB: 12.3% and DES 28.8% versus DCB 43.5%, *p* = 0.21. CABG still has a place for the treatment of ISR. In our population, 16 patients had undergone CABG surgery. We reported less MACEs during follow-up in the group of patients treated with CABG compared with patients treated with DES or DEB. Moustapha et al. [14] followed up for 2 years 510 symptomatic patients with ISR treated with various percutaneous therapies or CABG. The 2-year mortality was identical in both groups. However, the rate of target vessel revascularization or target lesion revascularization was higher after percutaneous techniques (33% vs. 8%, respectively, *p* = 0.05) which is similar to our results.

In our study, 16 patients (12.7%) received medical treatment alone. They were patients with multi-vessel disease not accessible for PCI. They had also high surgical risk, and CABG was not an option for them. Higher rates of MACEs were reported among these patients compared with patients treated with PCI or CABG (73.3% vs. 29.7%, *p* < 0.001). This can be explained by the persistence of non-revascularized coronary lesions.

Coronary angiography was the only way to diagnose and treat ISR. This can explain, in part, the high rate of secondary events in our series compared to those of the literature. Actually, the use of intra-coronary imaging for the diagnosis and treatment of ISR has been proven to significantly decrease the rate of secondary events as the commonest cause of DES restenosis is under sizing of the stent [15]. In fact, intra-coronary imaging allows the interventional cardiologist to assess correctly the deployment of the stent, and thus, he can optimize the angiographic result and prevent further ISR.

### Prognostic factors associated with worse outcome

#### Hypertension

In our study, we reported a higher rate of MACEs in hypertensive patients compared with normotensive patients (58% versus 23.2%; *p* = 0.049). Similarly, Kastrati et al. [16] showed that hypertension is independently correlated to the recurrence of angiographic ISR at 6 months with an OR of 1.21 (*p* = 0.009). However, Siontis et al. [17] did not identify a relationship between hypertension and ISR or the occurrence of MACE after treatment of ISR. Similarly, Kawamoto et al. [13] and Cassese et al. [18] did not find high blood pressure to be predictive of the occurrence of MACE after treatment of ISR. This may be explained by the poor control of blood pressure levels in our population.

#### Dyslipidemia

Dyslipidemia is a well-established cardiovascular risk factor. Many studies have suggested that elevated cholesterol, triglyceride, LDL-cholesterol and lipoprotein levels are correlated with a significant risk of ISR [19]. However, the limitation of these studies was that they have included a small number of patients who presented with acute coronary syndrome with variable lipid levels. Two randomized studies (Lovastatin Restenosis Trial [20] and FLARE [21]) confirmed the absence of a relationship between high lipid levels and the occurrence of MACE after the treatment of ISR. In our study, we found a higher rate of secondary events in the group of patients with high levels of LDL-cholesterol (38.7% vs. 18.9%; *p* = 0.02).

#### ISR of the LAD

Kastrati et al. [22] has shown that LAD stenting is an independent predictor of TLR after ISR treatment at 6 months. Our study is in line with these findings, where ISR of the proximal LAD is associated with a higher rate of MACEs (*p* = 0.04).

#### Absence of post-dilatation

Several studies have suggested that high optimization pressures do not affect clinical (occurrence of MACEs) and angiographic outcomes after stenting [23]. On the other hand, Rhee et al. [24] concluded that fully optimized angioplasty with prolonged inflation and sufficient post-dilation may play a key role in reducing TLR. In our study, we concluded that the absence of post-dilatation was an independent predictor of MACEs during follow-up after multivariate logistic regression [OR = 1.35, 95% CI 1.00–1.32, *p* = 0.04].

### Limits of the study

The main limit of our study was the lack of intra-coronary imaging devices (intravascular ultrasound or optical coherence tomography). These devices are essential to identify the mechanism and patterns of ISR. Fractional flow reserve was also lacking in our center. Severity of lesions was judged visually by the interventional cardiologist or referred for a stress test.

Moreover, it was a retrospective cohort study and the number of patients was limited compared to large published studies.

Furthermore, stent length has not been included in our study as it may represent a significant predictor of ISR.

## Conclusions

Our study suggests that ISR is a serious complication of coronary angioplasty with poor outcomes. Aggressive risk factors modification, exclusive use of DES and PCI optimization would lower significantly the burden of this complication.

## Data Availability

The data that support the findings of this study are available from medical files stored in the Farhat Hached hospital central archive. Restrictions apply to the availability of these data, which were used under license for the current study, and so are not publicly available. Data are however available from the authors upon reasonable request and with permission of the chief of cardiology department.

## Abbreviations

BMS: Bare-metal stent
CABG: Coronary artery bypass graft
CI: Confidence interval
DES: Drug-eluting stent
ISR: In-stent restenosis
LAD: Left anterior descending artery
MACE: Major adverse cardiac events
OR: Odds ratio
PCI: Percutaneous coronary intervention
